# Expression and Clinical Correlation Analysis Between Repulsive Guidance Molecule a and Neuromyelitis Optica Spectrum Disorders

**DOI:** 10.3389/fimmu.2022.766099

**Published:** 2022-02-03

**Authors:** Jinhua Tang, Xiaopeng Zeng, Jun Yang, Lei Zhang, Hang Li, Rui Chen, Shi Tang, Yetao Luo, Xinyue Qin, Jinzhou Feng

**Affiliations:** ^1^ Department of Neurology, The First Affiliated Hospital of Chongqing Medical University, Chongqing, China; ^2^ Department of Neurology, People's Hospital of Chongqing Hechuan, Chongqing, China; ^3^ Department of Biostatistics, School of Public Health and Management, Chongqing Medical University, Chongqing, China

**Keywords:** neuromyelitis optica spectrum disorders, repulsive Guidance Molecule a (RGMa), EDSS, AQP4, correlation analysis

## Abstract

**Objectives:**

This study sought to explore the expression patterns of repulsive guidance molecules a (RGMa) in neuromyelitis optica spectrum disorders (NMOSD) and to explore the correlation between RGMa and the clinical features of NMOSD.

**Methods:**

A total of 83 NMOSD patients and 22 age-matched healthy controls (HCs) were enrolled in the study from October 2017 to November 2021. Clinical parameters, including Expanded Disability Status Scale (EDSS) score, degree of MRI enhancement, and AQP4 titer were collected. The expression of serum RGMa was measured by enzyme-linked immunosorbent assay (ELISA) and compared across the four patient groups. The correlation between serum RGMa levels and different clinical parameters was also assessed.

**Results:**

The average serum expression of RGMa in the NMOSD group was significantly higher than that in the HC group (*p* < 0.001). Among the patient groups, the acute phase group exhibited significantly higher serum RGMa levels than did the remission group (*p < 0.001*). A multivariate analysis revealed a significant positive correlation between RGMa expression and EDSS score at admission, degree of MRI enhancement, and segmental length of spinal cord lesions. There was a significant negative correlation between the expression of RGMa in NMOSD and the time from attack to sampling or delta EDSS.

**Conclusions:**

The current study suggests that RGMa may be considered a potential biomarker predicting the severity, disability, and clinical features of NMOSD.

## Introduction

Neuromyelitis optica spectrum disorders (NMOSD) is a rare autoimmune demyelinating disorder of the central nervous system (CNS) associated with aquaporin-4 (AQP4) in which astrocytopathy is the primary pathology followed by neuroaxonal damage (1, 2). The prevalence of NMOSD varies from 0.5 to 4 per 100,000 people worldwide, 1 in 100,000 among Caucasians, 0.278 in 100,000 among Chinese individuals (3), and up to 10 in 100,000 among black individuals (4). Permanent disability rate is high among NMOSD patients, with approximately 35% of patients exhibiting severe visual impairment and 26% suffering from motor impairment (4, 5). Multiple episodes often lead to significant disabilities, incurring a heavy social and family burden. Early treatment can significantly reduce the disability rate and mortality, thus emphasizing the importance of early identification of NMOSD episodes and early treatment (6). AQP4 antibody can help diagnose NMOSD, but its titer level and disease severity do not always show a consistent trend or predict relapse (7–9).

Repulsive guidance molecule a (RGMa) is a glycosylphosphatidylinositol (GPI) anchored protein which guides axons and is widely involved in the development and pathology of the central nervous system (10). The binding of RGMa and its receptor neogenin can regulate axonal guidance, neuronal differentiation, and survival (11). Under pathological conditions, RGMa can affect functional recovery by inhibiting axon growth and participate in the pathogenesis of various CNS diseases, such as multiple sclerosis (MS), NMOSD, cerebral infarction (CI), spinal cord injury (SCI), Parkinson’s disease (PD), and epilepsy (12–18). Inhibiting RGMa can enhance the recovery of neural function, suggesting that RGMa may be a potential target for the treatment of CNS disorders (12–17). Humanized monoclonal anti-RGMa antibody has been reported to delay the onset of disease manifestations in rat models of NMOSD and alleviate disease severity (13). Our previous study had demonstrated that RGMa may play a critical role in reactive astrogliosis and glial scar formation (14), indicating that RGMa may be involved in the pathogenesis of NMOSD and may be used as a biomarker of disease activity.

However, the link between clinical parameters and RGMa in patients with NMOSD remains unknown. To assess whether RGMa may reflect the disease activity of NMOSD and the pathogenesis of NMOSD, we analyzed the correlation between serum RGMa levels and the clinical features of NMOSD.

## Methods

### Study Population

A total of 83 NMOSD patients and 22 age-matched healthy control patients from October 2017 to November 2021 were enrolled at The First Affiliated Hospital of Chongqing Medical University (Chongqing, China). Serum samples were collected and stored at -80°C. The diagnoses of all patients were reviewed, and only the patients who fulfilled the diagnostic criteria established by Wingerchuk et al. in 2015 were included (1). The following patients were excluded: those with malignant tumors, those with severe hepatic or renal insufficiency, and those without laboratory results of RGMa; the study was approved by the Ethics Committee of the First Affiliated Hospital of Chongqing Medical University. Informed consent was obtained from all the patients and HCs.

Medical records, laboratory data, and MRI findings were retrospectively assessed. Clinical information collected included age, sex, corresponding disease status (early acute phase, acute phase, or remission phase), number of relapses, total disease course, combined autoimmune disorders, Expanded Disability Status Scale (EDSS), serum levels of anti-AQP4 antibody, presentation of optic neuritis (ON), myelitis, magnetic resonance images (characteristic brain lesions on MRI, degree of MRI enhancement, length of spinal lesions, number of lesions on T2), delta EDSS (EDSS at discharge minus EDSS at admission), delta EDSS score after intravenous methylprednisolone (IVMP) (EDSS at the end of IVMP minus EDSS at admission when relapse occurred or first attack), and corticosteroid/immunomodulatory agent treatment history at the time of sampling. Time from attack to sampling refers to the disease duration from the time of the latest attack episode to sampling. According to the attack time, patients were divided into the early acute phase (episode ≤ 7 days), acute phase (7d<episode ≤ 30 days), and remission phase (episode > 30 days) (19). Neurological deficits were assessed using the EDSS score (20). The length of spinal lesions, characteristic brain lesions on MRI, and number of T2 lesions (including brain and spinal cord lesions) were counted using fluid-attenuated inversion recovery MRI scans (15). According to the enhancement degree of MRI lesions, patients were divided into no enhancement, mild enhancement, and marked enhancement groups. All patients underwent an MRI scan of the brain using a 3.0-T system (GE Medical Systems, Milwaukee, WI, USA) using an eight-channel phased-array head coil. Every contrast-enhancing lesion or hyperintensity on T_2_WI was delineated manually by two experienced neuroradiologists. The neuroradiologists were blinded to the characteristics of the study population, including brain MRI findings and clinical presentations. Cases in which neuroradiologists disagreed were reviewed and resolved by consensus.

### RGMa Measurement

Serum samples were centrifuged at 2500 rpm for 10 min at 4°C and stored thereafter at -80°C within 3 h of collection. The expression of serum RGMa was measured three times and the average value was obtained by enzyme-linked immunosorbent assay (ELISA) (R&D Systems Human RGM-A Assay kit; catalog No. DY2459-05). All sample measurements were carried out in a blinded fashion.

### Statistical Analysis

SAS 9.4 (SAS Institute Inc., Cary, North Carolina) was used for data analyses. Quantitative data of the normal distribution are presented as the mean ± SD. Two independent samples/paired t-tests and a variance analysis were used for comparisons between groups. Quantitative data of skewed distribution are presented as median and quartile intervals, and a Wilcoxon rank sum test was used for comparison between groups. Enumeration data were described by the number of cases and rates, and a chi-squared test was used for comparison between groups. The Pearson correlation coefficient describes the correlation between variables. A linear regression was used to assess the factors influencing the EDSS and RGMa. The mediation model was evaluated using Hayes’ model 4 in the PROCESS macro for SPSS, and a bootstrapping method with 2,000 resamples was used. Regression coefficients (βmult) were back-transformed to the original scale, and therefore reflected multiplicative effects. Age and sex were included in all the models for multivariate analysis. Bilateral p < 0.05, indicated that the difference was statistically significant.

## Results

### Participant Demographic Features and Clinical Data

A total 83 NMOSD patients and 22 healthy controls (HCs) were enrolled in the study. There was no significant difference in gender and age between the NMOSD and HC groups, as well as between the RGMa higher group and the RGMa lower group (all *p*>0.05). A total of 72 of the 83 patients (86.7%) tested positive for serum anti-AQP4 antibody. The median EDSS was 3.80 ± 1.73. Of the 83 patients with NMOSD, 37 had ON and 59 had myelitis.

### RGMa Serum Levels in Patients With NMOSD

The mean serum RGMa level was significantly higher in the general NMOSD patients than in the HCs (18800.32±8279.17 ng/ml vs. 8721.72 ± 9090.09 ng/ml, p < 0.001). Patients were divided into RGMa higher/lower group equally according to the RGMa level; results showed that RGMa higher group exhibited significantly higher EDSS (4.40 ± 1.64 vs. 3.19 ± 1.62, p <0.001), longer spinal lesion length (5.82 ± 2.08 vs. 3.57 ± 1.30, p<0.001) and higher delta EDSS score (-2.02 ± 1.13 vs. -0.65 ± 0.86, p<0.001) compared to the RGMa lower group (**Table 1**).

**Table 1:** Clinical data of patients with NMOSD.

	NMOSD (N = 83)	RGMa lower group (N = 41)	RGMa higher group (N = 42)	P value
Age (mean±Std)	38.94±15.30	41.97±15.48	35.98±14.70	0.074
Sex (Female,%)	63 (75.9)	32 (73.8)	31 (78)	0.652
Time from attack to sampling (median, IQR, day)	10 (5,26)	25 (7.5,55)	6 (4,10)	<0.001
Number of episodes (median, IQR)	2 (2,3.5)	2 (2,3)	2.5 (1.5,4)	0.009
EDSS at admission(mean±Std)	3.80±1.73	3.19±1.62	4.40±1.64	0.001
EDSS at discharge(mean±Std)	2.46±1.60	2.66±1.63	2.25±1.59	0.275
Delta EDSS	-1.32±1.21	-0.65±0.86	-2.02±1.13	<0.001
Serum levels of RGMa (mean±Std, ng/ml)	18800.32±8279.17	11746.78±3362.01	27382.85±6181.19	0.001
Length of spinal cord lesions (mean±Std)	4.72±2.07	3.57±1.30	5.82±2.08	<0.001
Number of lesions on T2 (median, IQR)	2 (2,6)	3 (1.5,5)	4.5 (3,7.5)	0.241

EDSS, Expanded Disability Status Scale; IQR, interquartile range;

Delta EDSS score (EDSS at discharge minus EDSS at admission).

**Figure 1:**
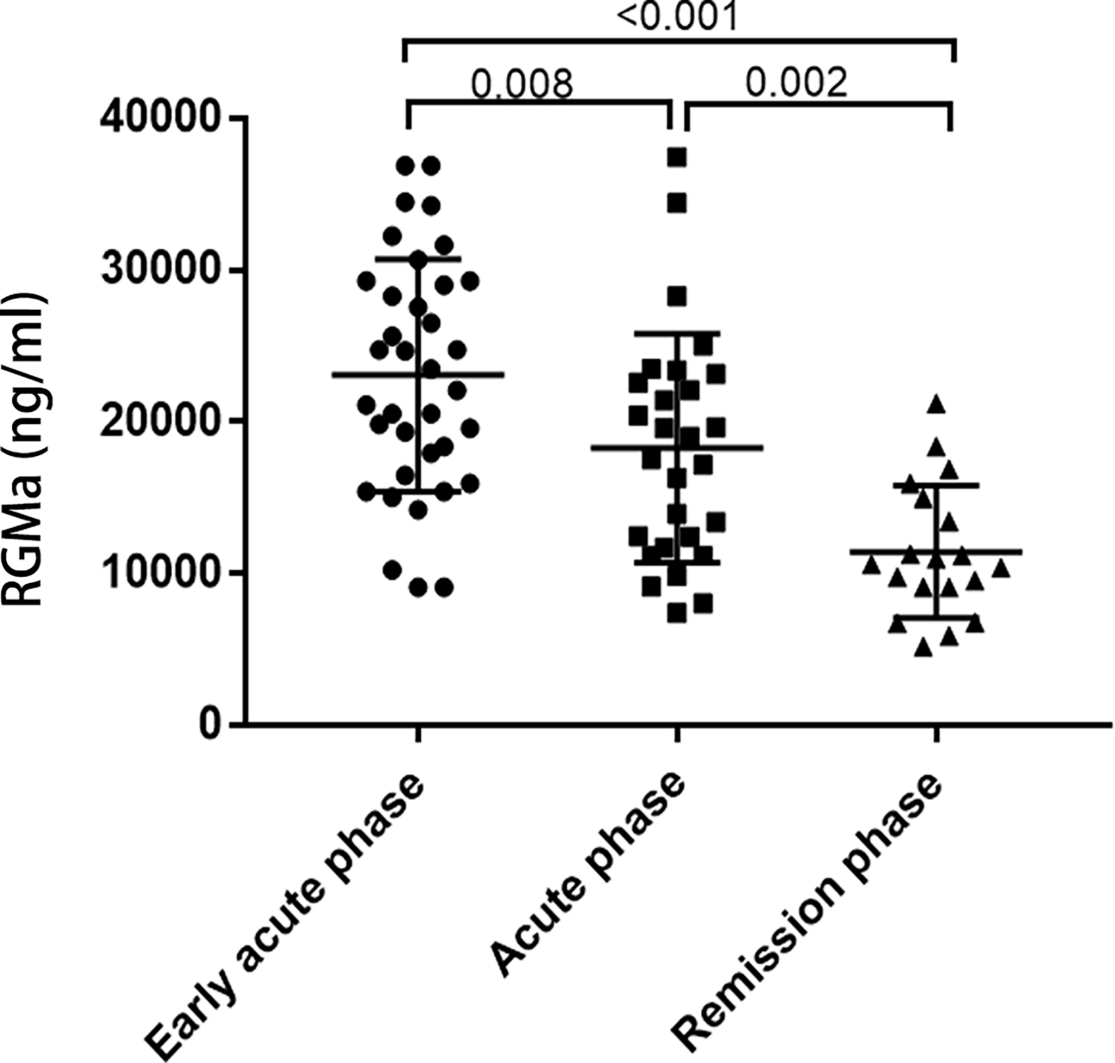
Comparison of serum RGMa levels according to the disease phase.

RGMa expression levels were upregulated in the early acute phase of NMOSD compared to acute phase (25078.83 ± 7649.24 ng/ml vs. 15523.17 ± 7236.70 ng/ml, p=0.008). The expression of RGMa was significantly higher in the acute group than the chronic group (12356.16 ± 4362.56 ng/ml, p=0.002) (**Figure 1**).

**Figure 2:**
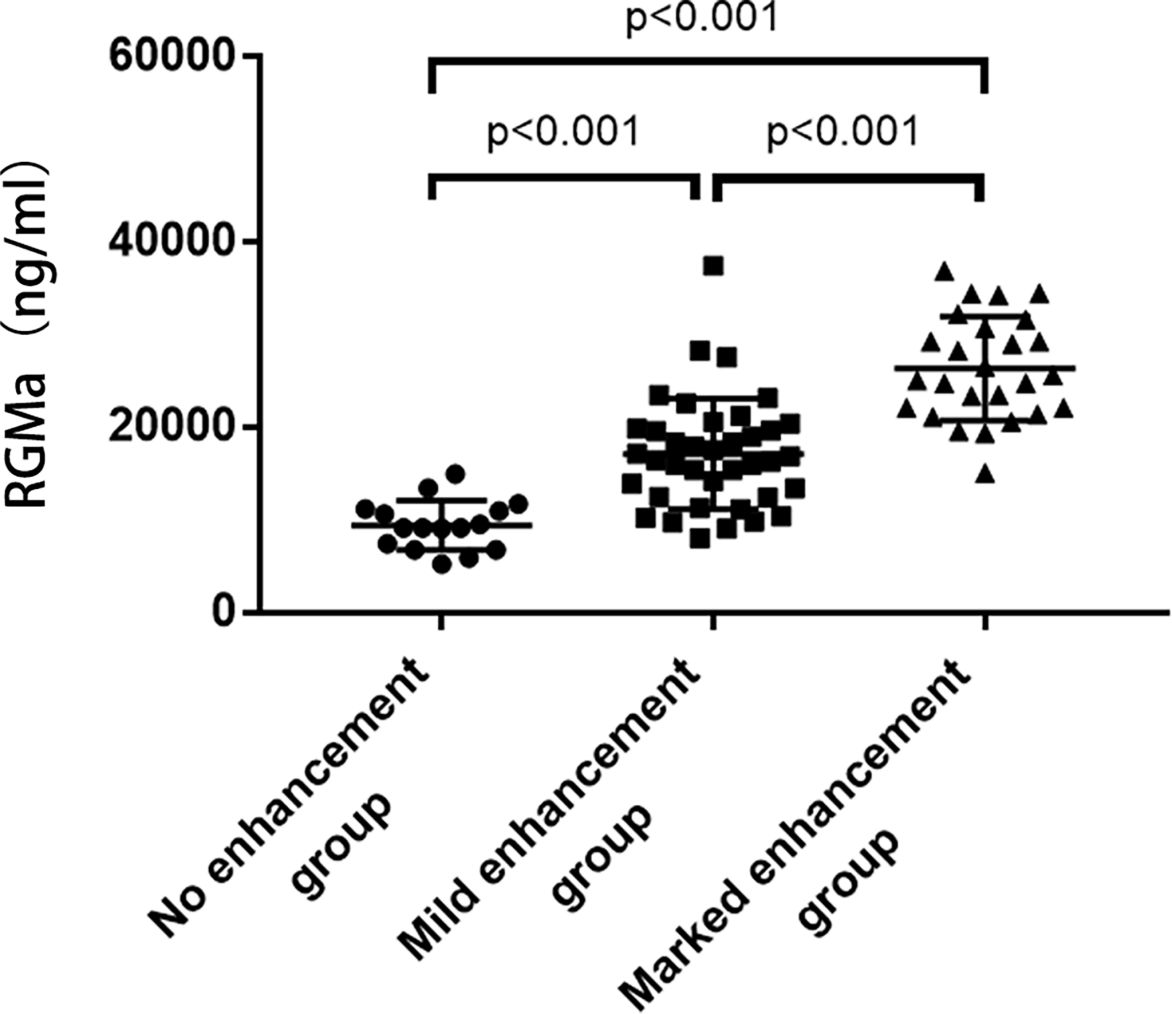
Comparison of serum RGMa levels according to MRI enhancement.

NMOSD patients were divided into three groups according to the degree of MRI enhancement, corresponding to the no enhancement group (n=16), mild enhancement group (n=38), and marked enhancement group (n=26). The expression of serum RGMa in marked enhancement group was significantly higher compared to mild enhancement group (26058.91 ± 5593.62 ng/ml vs. 16137.12 ± 5972.77 ng/ml, p<0.001). The expression of serum RGMa in the mild enhancement group was also significantly higher than that in the no enhancement group (16137.12 ± 5972.77 ng/ml vs. 9443.21 ± 2678.64 ng/ml, P<0.001) (**Figure 2**).

### Correlation of Serum Levels of RGMa With Clinical Parameters in NMOSD

As represented in **Table 2**, a significant positive correlation was observed between serum RGMa expression in NMOSD and EDSS at admission (r = 0.521, p <0.001), degree of MRI enhancement (r=0.757, p < 0.001), and length of spinal lesions (r=0.476, p < 0.001). There was a significant negative correlation between the expression of RGMa in NMOSD and time from attack to sampling (r=-0.444, P < 0.001) or delta EDSS (r=-0.640, p<0.001). Moreover, a multivariate model analysis showed that ESDD at admission, delta EDSS, degree of MRI enhancement, and length of spinal lesions were dependent factors that affected RGMa levels after adjusting for age and sex (p=0.035, p=0.009, p<0.001, p=0.014) (**Table 3**).

**Table 2:** Correlation of serum levels of RGMa with clinical parameters in NMOSD.

Variables	R	P value	R^2^
Age	-0.162	0.144	0.0262
Sex	-0.024	0.827	0.0006
Time from attack to sampling	-0.444	<0.001	0.1971
Number of episodes	-0.088	0.436	0.0077
Total disease course	-0.123	0.277	0.0151
EDSS at admission	0.521	<0.001	0.2714
EDSS at discharge (mean±Std)	0.019	0.872	0.0004
Delta EDSS	-0.640	<0.001	0.4096
AQP4 titer	-0.12	0.352	0.0140
Degree of MRI enhancement	0.757	<0.001	0.5731
Number of lesions on T2	0.169	0.179	0.0286
Length of spinal lesions	0.476	<0.001	0.2266

Delta EDSS score (EDSS at discharge minus EDSS at admission).

**Table 3:** Univariate and multivariate models testing the correlations between RGMa levels and clinical parameters in patients with NMOSD.

	Univariate			Multivariate		
	β	95%CI	p	β	95%CI	p
Age	3.612	(-205.65,30.45)	0.09	3.612	(-76.49,83.71)	0.929
Sex						
Female	–					
Male	-469.02	(-4721.68,3783.64)	0.83	-1683.055	(-4481.19,1115.08)	0.234
EDSS at admission	2503.87	(1585.81,3421.94)	<0.001	906.529	(65.88,1747.18)	0.035
Delta EDSS	-4341.10	(-5574.83,-3107.37)	<0.001	-1902.658	(-3310.286-495.029)	0.009
Time from attack to sampling	-56.20	(-81.27,-31.14)	<0.001	1.711	(-19.48,22.90)	0.872
Degree of MRI enhancement	8546.90	(6882.68,10211.12)	<0.001	6986.044	(4865.71,9106.38)	<0.001
Length of spinal lesions	2191.23	(1376.10,3006.36)	<0.001	861.077	(183.96,1538.20)	0.014

Delta EDSS score (EDSS at discharge minus EDSS at admission).

**Figure 3:**
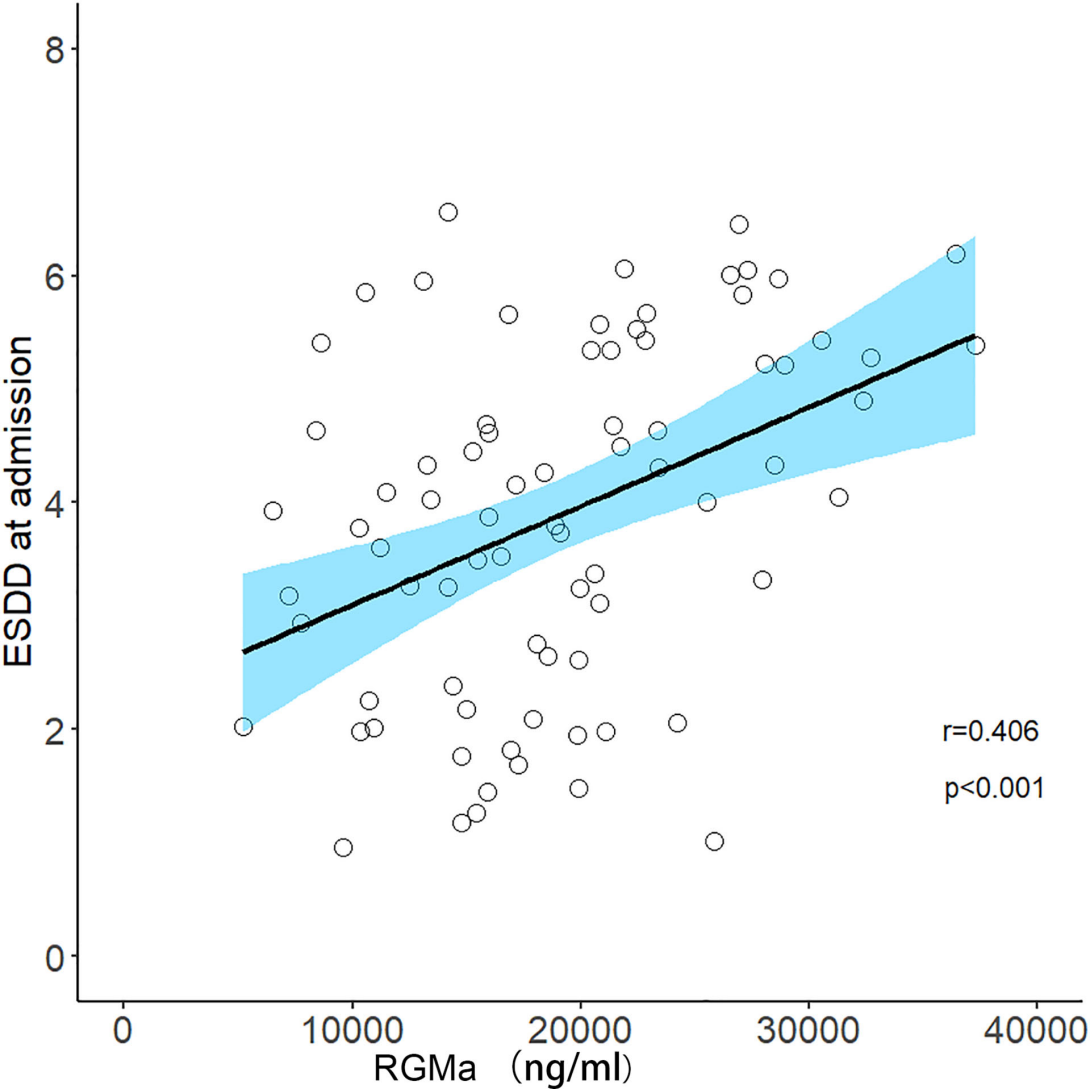
Correlation between serum RGMa level and EDSS score at admission in NMOSD after adjusting for sex, age, total disease course, time from attack to sampling, and number of episodes.

**Figure 4:**
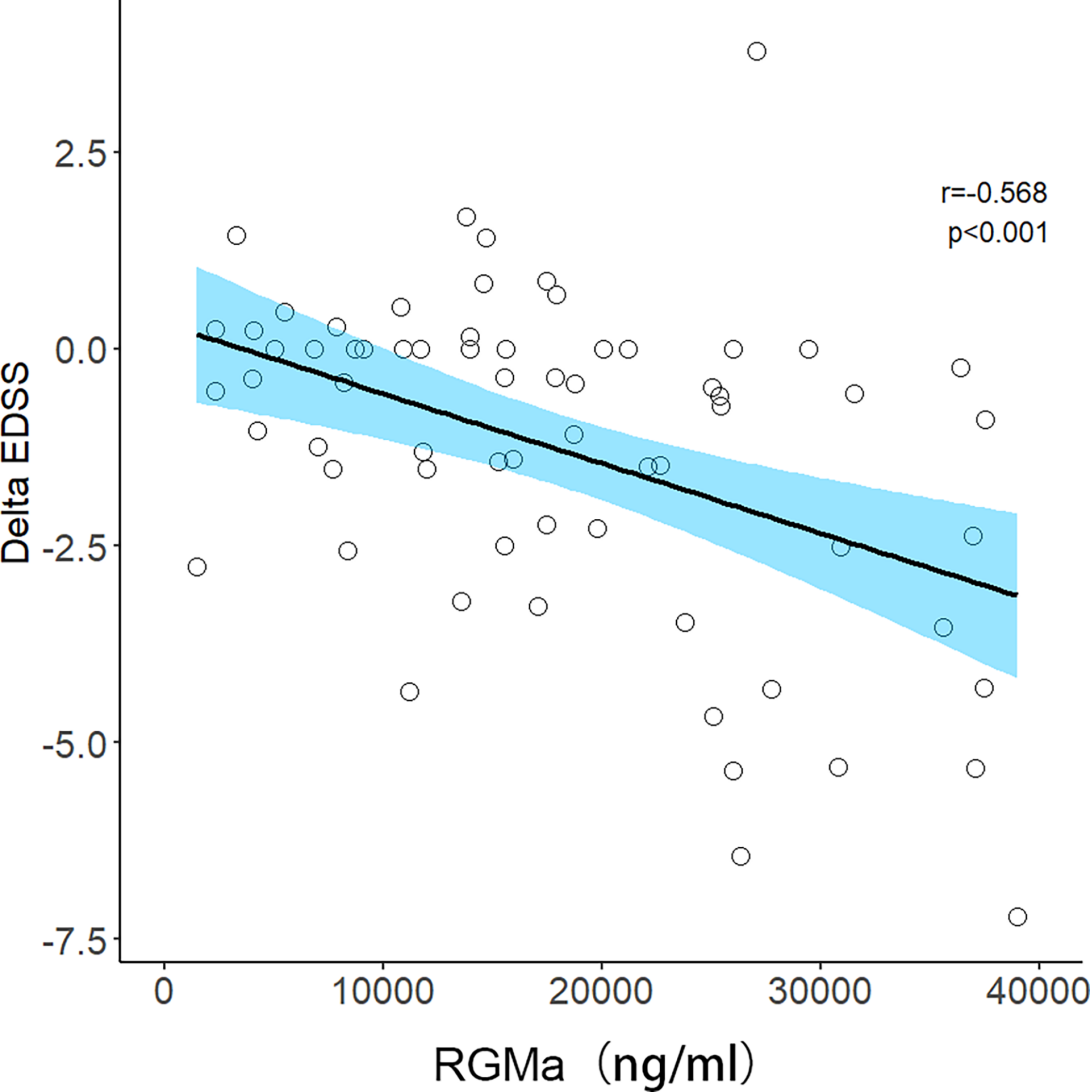
Correlation between serum RGMa level and Delta EDSS score in NMOSD after adjusting for sex, age, total disease course, time from attack to sampling, and number of episodes.

After adjusting for sex, age, total disease course, time from attack to sampling, number of episodes, the positive correlation observed between the serum RGMa expression in NMOSD and EDSS score at admission or negative correlation between RGMa expression and delta EDSS remained significant (r = 0.406, p <0.001, **Figure 3**; r=-0.568, p<0.001, **Figure 4**).

Paired t-test results demonstrated that RGMa significantly decreased after IVMP compared to before IVMP treatment in early acute phase patients (P=0.02) and was consistent with delta EDSS after IVMP. In terms of relapse, either myelitis or optic neuritis was independently associated with higher serum RGMa levels in the early acute phase (before acute phase treatment). Immunosuppressive/corticosteroid treatment was not an independent factor that influenced the levels of RGMa by comparing the first episode and non-first episode patients in early acute phase patients (**Table 4**).

**Table 4:** Comparison of RGMa level according to different clinical parameters.

	RGMa level	t	*P* value
Relapse in ON (n=9)	19196.78±3871.84	2.942	0.007
Non ON relapse (n=19)	26588.45±7030.32		
Relapse in myelitis (n=15)	29440.35±4123.50	7.078	<0.001
Non myelitis relapse (n=13)	18161.80±4298.86		
Before IVMP (n=20)	23870.2±7769.62	3.993	0.02
After IVMP (n=20)	17545.2±5977.55		
Delta EDSS after IVMP	1.16±1.10	-6.788	<0.01
First episode (n=14)	26747.23±5306.56	1.556	0.133
Non first episode (corticosteroid/ immunosuppressive agent) (n=15)	22676.01±8514.95		

A paired t-test was used for the two groups before and after IVMP treatment in the early acute phase; A independent t-test was performed for the other two groups.

### Mediating Effects of the Degree of MRI Enhancement and Time From Attack to Sampling in NMOSD Patients With Respect to RGMa Serum Levels and EDSS Score at Admission

As **Table 5** represents, for the path analysis of the mediation model, adjusting for age, gender, total disease course, and number of episodes, serum levels of RGMa were directly related to EDSS score at admission (β= 0.209, p < 0.01), and indirectly related to EDSS score at admission *via* degree of MRI enhancement, with indirect effects of β=0.283 (95% CI = [0.123, 0.456], p <0.01). The proportion of the mediating effect in the total effect was 57.52% (0.283/0.492).

**Table 5:** The mediating effects of degree of MRI enhancement between serum RGMa level and the EDSS score on admission.

Effect	β	LLCI	ULCI
**Direct effect**			
Serum levels of RGMa to EDSS at admission	0.240**	0.084	0.396
Serum levels of RGMa to EDSS at admission^△^	0.209**	0.046	0.373
**Indirect effect**			
Serum levels of RGMa to degree of MRI enhancement and EDSS at admission	0.258**	0.116	0.405
Serum levels of RGMa to degree of MRI enhancement and EDSS at admission△	0.283**	0.123	0.456
**Total effect**			
Serum levels of RGMa to EDSS at admission	0.499***	0.298	0.699
Serum levels of RGMa to EDSS at admission^†^	0.492***	0.284	0.701

^†^Adjusted for age, sex, total disease course, and number of episodes.

β=standardized coefficient; LLCI=lower limit of the 95% confidence interval. ULCI=upper limit at 95% confidence interval.

**P < 0.01,***P < 0.001.

As **Table 6** represents, serum levels of RGMa were directly related to EDSS score at admission (β= 0.386, p <0.001), and indirectly related to EDSS score at admission *via* time from attack to sampling, with indirect effects of β=0.127 (95% CI = [0.064,0.204], p <0.05). The proportion of mediating effect in the total effect was 24.76% (0.127/0.513).

**Table 6:** The mediating effects of time from attack to sampling between serum RGMa level and EDSS score on admission.

Effect	β	LLCI	ULCI
**Direct effect**			
Serum levels of RGMa to EDSS at admission	0.396***	0.197	0.595
Serum levels of RGMa to EDSS at admission^△^	0.386***	0.181	0.592
**Indirect effect**			
Serum levels of RGMa to Time from attack to sampling and EDSS at admission	0.118*	0.064	0.193
Serum levels of RGMa to Time from attack to sampling and EDSS at admission^△^	0.127*	0.064	0.204
**Total effect**			
Serum levels of RGMa to EDSS at admission	0.513***	0.317	0.709
Serum levels of RGMa to EDSS at admission^†^	0.513***	0.311	0.715

^†^Adjusted for age, sex, total disease course, and number of episodes.

β=standardized coefficient; LLCI=lower limit of the 95% confidence interval. ULCI=upper limit at 95% confidence interval.

*P < 0.05, ***P < 0.001.

## Discussion

Scientists have sought to explore biomarkers reflecting the activity of NMOSD, such as neurofilament light (NfL), GFAP, and tau, but none have been generally recognized as biomarkers for predicting disease severity and clinical features. The current study is the first to explore the clinical value of plasma RGMa levels as a biomarker in patients with NMOSD. Enhanced magnetic resonance imaging is widely used to monitor the disease activity of NMOSD, but the accumulation of gadolinium contrast agents has raised safety concerns (21, 22). In addition, some studies have revealed that approximately 50% of patients with NMOSD still present with enhanced lesions one month after the onset of IVMP therapy, thereby suggesting that enhanced magnetic resonance imaging cannot accurately reflect the disease activity of NMOSD patients in the late stage of an attack (23).

Our previous study had demonstrated that RGMa may play a critical role in reactive astrogliosis and glial scar formation (14). This study showed that plasma RGMa levels in NMOSD patients were significantly higher than those in healthy controls. Considering that NMOSD are primarily astrocytopathy, suggesting that RGMa may be linked to the pathogenesis of NMOSD.

Many studies have demonstrated that RGMa may be involved in the pathogenesis of multiple sclerosis (MS) *via* a variety of mechanisms, such as by promoting demyelination of the central nervous system, inhibiting axon regeneration, resulting in the abnormal signal transduction of immune cells, inhibiting angiogenesis, and regulating BBB permeability (24–33). Fully humanized anti-RGMa mAb are currently being assessed in the context of double-blind, placebo-controlled, randomized clinical trials (34). However, the specific mechanisms by which RGMa affect the pathogenesis of NMOSD patients remain unclear.

Previous studies have reported that plasma RGMa is inversely related to delta EDSS in patients with MS (12). We also found that plasma RGMa levels were negatively correlated with delta EDSS and delta EDSS scores following IVMP in NMOSD. Moreover, the level of RGMa was positively correlated with the admission EDSS score at admission when relapsed, indicating that RGMa is associated with neurological impairment. Even though RGMa higher group showed a higher delta EDSS score compared to RGMa lower group, it still hard to considering RGMa level itself as a dependent predictor to NMOSD prognosis, but the variation of RGMa level related to treatment is more valuable. Those combined results indicate that plasma RGMa may be used as an index to predict therapeutic effects in the acute phase.

Currently, the onset time and degree of MRI enhancement can reflect disease activity to some extent (21–23). The destruction of the blood–brain barrier (BBB) is an important pathological process in the acute phase of NMOSD, and the degree of MRI enhancement can reflect the degree of BBB damage in NMOSD patients (35, 36). We have revealed that RGMa expression consistent with the onset time according to the acute phase group had significantly higher serum RGMa expression level than that in the remission group. Furthermore, RGMa serum expression level in the marked enhancement group was the highest among the mild enhancement group and the lowest in the no enhancement group. We conclude that RGMa expression was negatively correlated with the time from attack to sampling and positively correlated with the degree of magnetic resonance enhancement. These results demonstrate that RGMa may be a relatively useful biomarker for NMOSD disease activity in predicting the treatment of the acute phase. RGMa may be related to BBB permeability damage in NMOSD.

The length of spinal lesions is correlated with inflammatory activity and neurological impairment (21, 37) and is correlated with disease severity based on the admission EDSS score (38, 39). Our study demonstrated for the first time that RGMa levels were positively correlated with the length of spinal lesions on NMOSD MRI scans.

The mediation effect analysis revealed that RGMa was directly related to the admission EDSS score and indirectly related to the admission EDSS score through time from attack to sampling and degree of MRI enhancement. This highlights that RGMa may partially affect the degree of neurological impairment by affecting inflammatory activity and BBB permeability in NMOSD.

Our current study had a number of limitations. First, since it was carried out as a retrospective study, limited clinical data were collected, such as the lack of accurate quantitative indicators for BBB damage. Second, it was carried out as a single-center study with a relatively small sample size. Finally, this study lacked a long-term follow-up period with the patients to assess the relationship between RGMa and long-term prognosis of NMOSD. Last but not the least, MRI enhancement assessments were carried out by individuals instead of by using a more objective, quantitative algorithm, which may have compromised the MRI enhancement correlation analysis to a certain degree. In the future, we will try to carry out a number of multi-center and prospective studies to expand the sample size and further validate our research results. In addition, we will increase the volume of clinical data and carry out proof-of-concept experiments using animal models to further assess the pathogenesis of RGMa in NMOSD to guide clinical diagnosis and treatment.

## Conclusion

In summary, we found that the serum RGMa levels of NMOSD patients were significantly higher than those of healthy controls. The expression and clinical correlation analysis between RGMa suggested that RGMa may be considered as a relatively safe and quantitative potential biomarker for predicting the activity of NMOSD. We demonstrated that higher RGMa levels in patients with NMOSD were associated with more severe neurological deficits, longer segments of spinal cord lesions, shorter onset time, higher plasma AQP4 antibody titers, and more obvious lesion enhancement. This suggests that RGMa can reflect the disease activity of NMOSD and may be involved in the pathogenesis of NMOSD by affecting BBB permeability and AQP4; however, further studies are warranted.

## Data Availability Statement

The raw data supporting the conclusions of this article will be made available by the authors, without undue reservation.

## Ethics Statement

The studies involving human participants were reviewed and approved by Ethics Committee of the First Affiliated Hospital of Chongqing Medical University. The patients/participants provided their written informed consent to participate in this study.

## Author Contributions

All authors contributed to the manuscript and approved the submitted version. JT drafted the manuscript, XZ collected the sample and YL analysis, and interpretation of the data. XQ and JF designed the study and revised the manuscript.

## Funding

This work was supported by the National Natural Science Foundation of China to Jinzhou Feng (No. 81701191) and the National Key Clinical Specialties Construction Program of China.

## Conflict of Interest

The authors declare that the research was conducted in the absence of any commercial or financial relationships that could be construed as a potential conflict of interest.

## Publisher’s Note

All claims expressed in this article are solely those of the authors and do not necessarily represent those of their affiliated organizations, or those of the publisher, the editors and the reviewers. Any product that may be evaluated in this article, or claim that may be made by its manufacturer, is not guaranteed or endorsed by the publisher.
